# Structural and Enzymatic Characterization of ABgp46, a Novel Phage Endolysin with Broad Anti-Gram-Negative Bacterial Activity

**DOI:** 10.3389/fmicb.2016.00208

**Published:** 2016-02-26

**Authors:** Hugo Oliveira, Diana Vilas Boas, Stéphane Mesnage, Leon D. Kluskens, Rob Lavigne, Sanna Sillankorva, Francesco Secundo, Joana Azeredo

**Affiliations:** ^1^Centre of Biological Engineering, Laboratório de Investigação em Biofilmes Rosário Oliveira, University of MinhoBraga, Portugal; ^2^Krebs Institute, Department of Molecular Biology and Biotechnology, University of SheffieldSheffield, UK; ^3^Laboratory of Gene Technology, KU LeuvenLeuven, Belgium; ^4^Istituto di Chimica del Riconoscimento Molecolare – Consiglio Nazionale delle RicercheMilano, Italy

**Keywords:** *Acinetobacter baumannii*, phage endolysins, mass spectrometry, circular dichroism, antibacterial activity

## Abstract

The present study demonstrates the antibacterial potential of a phage endolysin against Gram-negative pathogens, particularly against multidrug resistant strains of *Acinetobacter baumannii*. We have cloned, heterologously expressed and characterized a novel endolysin (ABgp46) from *Acinetobacter* phage vb_AbaP_CEB1 and tested its antibacterial activity against several multidrug-resistant *A. baumannii* strains. LC-MS revealed that ABgp46 is an *N*-acetylmuramidase, that is also active over a broad pH range (4.0–10.0) and temperatures up to 50°C. Interestingly, ABgp46 has intrinsic and specific anti-*A. baumannii* activity, reducing multidrug resistant strains by up to 2 logs within 2 h. By combining ABgp46 with several organic acids that act as outer membrane permeabilizing agents, it is possible to increase and broaden antibacterial activity to include other Gram-negative bacterial pathogens. In the presence of citric and malic acid, ABgp46 reduces *A. baumannii* below the detection limit (>5 log) and more than 4 logs *Pseudomonas aeruginosa* and *Salmonella typhimurium* strains. Overall, this globular endolysin exhibits a broad and high activity against Gram-negative pathogens, that can be enhanced in presence of citric and malic acid, and be used in human and veterinary medicine.

## Introduction

Gram-negative (G^-^) pathogenic bacteria remain a global human health concern as they are common causes of foodborne, environmentally acquired, and zoonotic infectious diseases (Chopra et al., 2008; Scallan et al., 2011). This problem is exacerbated by their ability to display intrinsic [e.g., low outer membrane (OM) permeability] or acquired antibiotic resistant mechanisms (e.g., horizontal transfer of resistance genes; Bonomo and Szabo, 2006). *Acinetobacter baumannii* and *Pseudomonas aeruginosa* cause a wide range of nosocomial diseases (including wound, skin infections) and are characteristic strains for which resistance has occurred against and spread to nearly all antibiotics available. The development and spread of multidrug resistant pathogens have instigated and intensified the interest in alternative antimicrobials.

Bacteriophage (phages) lytic enzymes, called endolysins, are proteins synthesized at the end of the phage lytic cycle to destroy the cell wall peptidoglycan (PG) to release viral progeny (Fischetti, 2005). Endolysins have either a single catalytic domain (globular structure) responsible for the PG digestion by glycosidase, amidase or peptidase activity, typically observed in phages infecting G^-^ hosts, but can contain a second domain [modular structure, an architecture more prominent in Gram-positive (G^+^)-like endolysins] that aids substrate recognition and binding (Oliveira et al., 2012). Recent *in silico* analysis illustrated the large diversity and complexity of endolysins that can comprise up to four domains in the same coding sequence, have 24 different types of catalytic and 15 binding domains, and 89 possible architectural organizations (Oliveira et al., 2013). In view of this tremendous diversity, endolysins have received relatively little attention, especially in terms of more fundamental aspects such as their conformation analysis as well as their its anti-G^-^ activity.

Due to their biotechnological potential of endolysins including their unique ability to rapidly cleave the bacterial PG through external application and the absence of reported resistance development, endolysins have sparked the interest as alternatives for existing antibiotics (Nelson et al., 2001). However, a review of the literature still demonstrates an overwhelming and almost exclusive application of endolysins in combating G^+^ pathogens (Fischetti, 2010). This has been shown, for example, on methicillin-resistant and multidrug-resistant *Staphylococcus aureus* (endolysins LysK and MV-L, respectively; O’Flaherty et al., 2005; Rashel et al., 2007), on vancomycin-resistant *Enterococcus faecalis* and *E. faecium* (endolysin PlyV12; Yoong et al., 2004). The limited number of publications against G^-^ bacteria is explained by permeabilization issues due to the presence of the OM that hinders the action of externally added endolysins (Vaara, 1999). To date, only a few endolysins were successfully reported to kill G^-^ cells (Lood et al., 2015), mostly combining endolysin activity with the chelating effect of EDTA to permeabilize the OM and, more recently, by novel engineering strategies, for specific applications (Lim et al., 2012; Walmagh et al., 2012; Briers et al., 2014a,b; Lood et al., 2015). Recently, the application of endolysins in combination with weak acids (citric and malic acid), also proved to be successful in eliminating a broad range of G^-^ pathogens (Oliveira et al., 2014).

This study describes the isolation of a phage-encoded endolysin (ABgp46) originating from an *Acinetobacter* phage vb_AbaP_CEB1. ABgp46 was fully characterized in terms of its primary, secondary and tertiary structure under different physiological conditions giving novel insights into this class of proteins. Its antibacterial action was studied by combining the endolysin with several organic acids (citric, malic, lactic, benzoic, and acetic acid) against antibiotic resistant G^-^ bacteria. This work demonstrates that weak acids are suitable OM permeabilizers (OMPs), enabling ABgp46 to reach the PG and kill the cells.

## Materials and Methods

### Bacterial Strains, Media, and Chemicals

Bacterial strains were acquired either from the American Type Culture Collection (*S. typhimurium* ATCC 19585, *P. aeruginosa* ATCC 15692, and *Klebsiella oxytoca* ATCC 131821) or from the Spanish Type Culture Collection (*Escherichia coli* O157:H7 CECT 47821). Clinical *Acinetobacter* isolates are multi-resistant and were kindly provided by the Hospital of Braga with patterns of antibiotic resistance given for each strain. All strains were grown in Lysogeny Broth (LB; Liofilchem) at 37°C and 120 rpm. For transformation, chemically competent *E. coli* TOP10 and *E. coli* BL21(DE3) cells (Invitrogen) were prepared for cloning and recombinant protein expression respectively. The *Acinetobacter* phage vb_AbaP_CEB1 (encoding the ABgp46 endolysin) was isolated from waste eﬄuents and belongs to the Centre of Biological Engineering phage collection (Braga, Portugal). This phage belongs to the *Podoviridae* family and is a T7 likeviruses EDTA was acquired from Pronalab while isopropyl-β-D-thiogalactopyranoside (IPTG), HEPES and the organic acids were purchased from Sigma-Aldrich.

### Cloning, Recombinant Protein Expression, and Purification

Phage DNA was extracted from concentrated purified phage lysates using the phenol:chloroform extraction method (Sambrook et al., 2001). Afterward, the ABgp46 ORF was amplified from the phage genomic DNA using Phusion High-Fidelity DNA Polymerase (NEB) and a set of specific primers (forward: 5-GGCAGCCATATGATTCTGACTAAAGACGGGTTTAG and reverse: 5-GCAGCCGGATCCCTATAAGCTCCGTAGAGCGC, with the NdeI/BamHI restriction endonuclease sites underlined). Next, the amplicon was purified (DNA Clean & Concentrator-5k, Zymo Research), digested using NdeI and BamHI enzymes (NEB), and cloned in the pET15b expression vector (Novagen) with an N-terminal His6-tag. The insert was confirmed by Sanger sequencing (Eurofins). The sequence of ABgp46 was deposited in GenBank under accession no. KP998152.

*Escherichia coli* BL21(DE3) was transformed with the pET15b-ABgp46 vector and the endolysin produced as follows: cells were grown in 200 mL LB (supplemented with 100 μg/mL of ampicillin) to an OD_600__nm_ of 0.6 (approximately 4 h, 120 rpm at 37°C; ES-20/60), and recombinant protein expression induced with 0.5 mM IPTG at 16°C, 120 rpm overnight. The culture was then centrifuged (9500 × *g*, 30 min), and the cells disrupted by suspending the pellet in 1/25 volumes of lysis buffer (20 mM NaH_2_PO_4_, 0.5 M NaCl/NaOH, pH 7.4), followed by three cycles of freeze-thawing (-80°C to room temperature). Maintaining the sample on ice, cells were further disrupted by sonication (Cole-Parmer, Ultrasonic Processors) for 8–10 cycles (30 s pulse, 30 s pause). Insoluble cell debris was removed by centrifugation (9500 × *g*, 30 min, 4°C) and the supernatant was collected, filtered (0.22 μm filters, GE Healthcare) and applied to Ni^2+^-NTA resin stacked in HisTrapTM HP 1 mL columns (GE Healthcare) for purification, using a imidazole gradient (25–300 mM). Eluted protein fractions were visualized by standard denaturation SDS-PAGE gels, and only protein fractions with purity >95% were dialyzed in 10 mM PBS at pH 7.2 (using Maxi GeBAflex-tube Dialysis Kit – Gene Bio-Application Ltd). The protein was quantified using the BCA TM Protein Assay Kit (Thermo Scientific).

### Endolysin Activity Characterization

#### Detection and Quantification of Muralytic Activity

The assessment of the ABgp46 muralytic (PG degrading) activity was performed by visualizing inhibition spots on G^-^ bacteria lawns, as described elsewhere (Schuch et al., 2009). Briefly, 100 μL of *Acinetobacter* strains grown to an OD_600__nm_ of 0.6 were spread over the surface of LB agar and grown overnight at 37°C. Formed bacterial lawns were subsequently treated with chloroform vapors for 30 min to induce OM permeabilization, after which a 30-μL drop of purified ABgp46 was spotted. After a few minutes, the visualization of inhibition halos determined the presence of a endolysin activity, for its ability to degrade the exposed PG.

The muralytic activity of ABgp46 was quantified on permeabilized *P. aeruginosa* cells following treatment with chloroform/Tris-HCl to allow the protein to reach its PG substrate (Lavigne et al., 2004). Briefly, mid-exponential *P. aeruginosa* strains were centrifuged (4600 × *g*, 30 min at 4°C), suspended in the upper layer of chloroform-saturated 50 mM Tris-HCl, pH 7.7 solution and incubated for 45 min at 120 rpm. Following incubation, bacterial protoplasts were collected (4000 × *g*, 15 min, 4°C), washed and suspended in 80 mM of KH_2_PO_4_/K_2_HPO_4_ buffer (pH 7.2) then adjusted to an OD_600__nm_ of 1.2. Bacterial protoplasts were stored at -20°C prior to use. To measure enzymatic activity, 30 μL of enzyme (0.125, 0.25, 0.5, 1, 2, 4, and 8 μM) were added to 270 μL of permeabilized *P. aeruginosa* cells. The resulting decrease in optical density was measured spectrophotometrically (BIO-TEK^®^, Synergy HT Microplate Reader) for up to 30 min of reaction with readings taken every 30 s. To measure the pH dependence, chloroform/Tris-HCl permeabilized OM *P. aeruginosa* cells were suspended in a universal pH buffer (10 mM KH_2_PO_4_, 10 mM Na-citrate and 10 mM H_3_BO_4_), with a pH adjusted between 4.0 and 10.0. Obtained OD values were adjusted for the contribution of the negative control (PBS treated). The muralytic activity was calculated based on the best linear regression of the saturation curve and expressed in units/μM according to a validated method explained in detail elsewhere (Briers et al., 2007).

#### Stability

The enzyme stability was tested using similar turbidity tests as described previously (Oliveira et al., 2014). Enzyme kinetics experiments were performed using 2 μM of ABgp46 stored at 4°C for 2 months, or heated at different temperatures of 20, 30, 40, 50, and 60°C for 30 min in a MJ Mini BIO-RAD Thermocycler, in the universal buffer at optimal pH. The residual muralytic activity of each sample relative to the activity of the unheated reference sample at time 0 (=100% activity) was determined.

#### Determination of the PG Cleavage Site

The ABgp46 cleavage site was determined using *E. coli* BW25113 Δ*lpp* PG sacculi as a substrate. PG was extracted from exponentially growing *E. coli* cells as previously described using boiling SDS (Glauner, 1988). Five hundred μg of pure PG was digested overnight with 0.6 mg/ml of ABgp46 at 37°C in 25 mM of phosphate buffer pH 6.0, supplemented with 0.1 mM of MgCl_2_ in a final volume of 100 μl. *E. faecalis* AtlA *N*-acetylglucosaminidase (0.6 mg/ml) and *S. globisporus N*-acetylmuramidase mutanolysin (Sigma-Aldrich; 500 μg/ml) were used as controls to digest 500 μg of *E. coli* PG in 25 mM Tris-HCl (pH 8.0) and 25 mM of phosphate buffer (pH 6.0), respectively. Soluble muropeptides were recovered by centrifugation (20,000 × *g*, 15 min, 20°C), reduced with 5 mg/ml of sodium borohydride and separated by reverse-phase HPLC (RP-HPLC) on a Hypersil aQ C_18_ column (3 μm; 2.1 by 200 mm; ThermoFisher Scientific) connected to an Agilent 6500 Series Q-TOF LC/MS System. Muropeptides were eluted at a flow rate of 0.25 ml/min with a 0 to 15% gradient applied between 6 and 40 min [Buffer A, 0.1% (v/v) formic acid in water; buffer B, 0.1% (v/v) formic acid in acetonitrile].

#### Endolysin Conformation Stability

##### Fluorescence measurements

The intrinsic fluorescence emission spectrum of the ABgp46 was measured over the 300–400 nm range using a Jasco FP-750 spectrofluorimeter equipped with a Peltier thermostat. An excitation wavelength of 295 nm was used to minimize the emission arising from tyrosine residues. Thermal denaturation was monitored by heating 2 μM protein in universal buffer with a constant rate of 2°C/min from 20 to 70°C. The variation of the fluorescence spectra was measured as a variation of Parameter A defined as the ratio of intensity of fluorescence (IF) at 360 and 325 nm (IF 360/IF 325). Parameter A was plotted as a function of temperature and fitted in Boltzmann sigmoidal curves.

##### Circular dichroism analysis

Endolysin circular dichroism (CD) spectra were recorded in triplicate in the Far-UV region (195–260 nm) using a J-1100 CD Spectrometer, Jasco, in the universal buffer (pH 4.0–10.0). Spectra were recorded at desired pH, from 20 to 70°C, with a thermal increase rate of 2°C/min. All CD spectra were baseline corrected and smoothed with the Spectra Analysis JASCO software. The intensity of the CD signal measured at 222 nm was plotted as a function of temperature and fitted in Boltzmann sigmoidal curves. In all cases, an enzyme concentration of 8 μM (0.18 mg/mL) and an optical path of 0.1 cm were used.

#### Antibacterial Assays

*In vitro* assays on planktonic cells were performed as described previously (Oliveira et al., 2014), with minor modifications. Mid-exponential phase cells (OD_600__nm_ of 0.6) suspended and 100-fold diluted in 10 mM HEPES/NaOH (pH 7.2), were prepared. Each culture (50 μL) was incubated for 2 h at room temperature with 25 μL of ABgp46 (final concentration of 2 μM) together with 25 μL of water or 25 μL or OMPs (EDTA, citric, malic, lactic, benzoic, or acetic acid) dissolved in water. Parallel experiments were also carried out supplementing the reaction with 5 mM of MgCl_2_ to evaluate the effect of free divalent cations in the solution. In all cases, negative controls were included by incubating 50 μL of cells with 25 μL of PBS (pH 7.2; replacing ABgp46) or with 25 μL water (instead of OMPs). After incubation, CFUs were counted in LB agar plates and the antibacterial activity quantified as the relative inactivation in logarithmic units [= log_10_ (*N*_0_/*N*_i_) with *N*_0_ = number of untreated cells (in the negative control) and *N*_i_ = number of treated cells counted after incubation]. Averages ± standard deviations for all experiments are given for *n* = 4 repeats.

## Results

### *In silico* Analysis of the Primary Structure

*Acinetobacter* phage vB_AbaP_CEB1 was previously isolated and sequenced (unpublished data). ORF 46 (referred to as ABgp46), encoding a 185-amino acid protein with a deduced molecular mass of 23.1 kDa, is predicted to act as a PG hydrolase with a HHpred output showing that ABgp46 belongs to the CAZY glycosidase family 19 (GH19; E-value = 2 × 10^-36^; Cantarel et al., 2009). GH19 represents a class of chitinases that cleaves the unbranched chains of *N*-acetyl glucosamine polymers, a structure uncommon in bacterial cell walls, but some enzymes that able to degrade the PG of G^-^ bacteria have also been shown (e.g., *Pseudomonas* phage OBP and *Salmonella* phage PVP-SE1 endolysins; Walmagh et al., 2012).

BlastP analyses showed that the protein has a high sequence similarity to four other predicted endolysins from *Acinetobacter* phages (phiAB1, phiAB3, ABP-01, and ABP-04). Amphipathic helices were identified between the amino acids 112 and 145 (**K**NPE**K**ALEPLIAIQIAI**K**GMLNGWFTGVGF**RRKR**), with positively charged amino acids shown in bold. The same sequence has been observed in the *A. baumannii* phage endolysin LysAB2, which has been shown to interfere with the *A. baumannii* OM (Lai et al., 2011). ABgp46 was produced as a recombinant protein in *E. coli* and purified under native conditions, yielding a soluble protein of 20.5 mg per liter of culture.

### Endolysin Muralytic Activity, pH Dependence, and Stability

**FIGURE 1:**
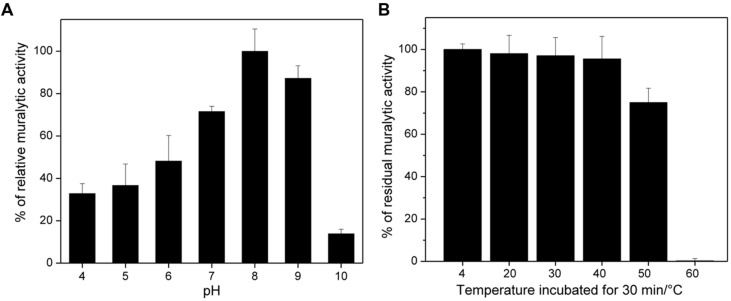
**pH and temperature effect on the ABgp46 activity. (A)** The pH dependence was measured as the slope of the OD_600__nm_/min curve, using *Pseudomonas aeruginosa* OM permeabilized cells as a substrate, suspended in a universal buffer (10 mM KH_2_PO_4_, 10 mM Na-citrate, and 10 mM H_3_BO_4_), and with pH adjusted from 4.0 to 10.0. **(B)** The stability was tested by heating the protein at different temperatures (20–60°C) for 30 min, and using OM permeabilized *P. aeruginosa* PAO1 cells at pH of 8.0. The residual activity shown, is expressed in percentage by comparing with ABgp46 stored at 4°C (=100% relative activity). Averages and standard deviations of four repeated experiments are shown.

ABgp46 was able to lyse OM compromised *A. baumannii* #2 lawn(phage host) and its activity calculated using turbidimetry assays. ABgp46 is active between of pH of 4.0–9.0 with an optimal between 8.0 and 9.0 (**Figure 1A**). At optimal pH, ABgp46 had a muralytic activity of 490 units/μM. In addition, ABgp46 remained fully active after 1 month at 4°C with a 25% decrease in activity observed after 30 min incubation at 50°C and complete inactivation after 30 min incubation at 60°C (**Figure 1B**).

### RP-HPLC and LC-MS Analysis of the Biochemical Activity of ABgp46

The PG bond cleaved by ABgp46 was identified by LC-MS (Supplementary Figures S1 and S2) using *E. coli* purified PG as a substrate. The muropeptide profiles corresponding to the digestion of *E. coli* PG by ABgp46, *E. faecalis* AtlA (an *N*-acetylglucosaminidase) and mutanolysin (an *N*-acetylmuramidase) were similar, suggesting that ABgp46 displays glycosyl hydrolase activity. MS analysis of the muropeptides corresponding to the major monomers solubilized (peaks 1–3 in Supplementary Figure S1) identified ions with *m/z* values of 942.415, 942.414, and 942.414, respectively, matching the theoretical value expected for a disacharride-tetrapeptide (942.414). To identify whether ABgp46 is an *N*-acetylmuramidase or an *N*-acetylglucosaminidase, we analyzed the fragmentation pattern of the ions with an *m/z* at 942.414 to identify the sugar moiety harboring a reducing group (Eckert et al., 2006). Major ions in peaks 1 (mutanolysin digestion) and 3 (ABgp46 digestion) both revealed a fragmentation event leading to the loss of a non-reduced GlcNAc residue (-203.078 atomic mass units, seen as a [M+H]^+^ adduct with *m/z* at 204.086 and 204.087) indicating that ABgp46 displays *N*-acetylmuramidase (lysozyme) activity. By contrast, the major ion in peak 2 (AtlA digestion) revealed a fragmentation event leading to the loss a reduced GlcNAc residue (-223.106 atomic mass units, seen as a [M+H]^+^ adduct with an *m/z* at 224.113) expected for an *N*-acetylglucosaminidase activity (Supplementary Figure S2). Collectively, these results demonstrate that ABgp46 displays *N*-acetylmuramidase activity.

### Conformational Analysis of ABgp46

**FIGURE 2:**
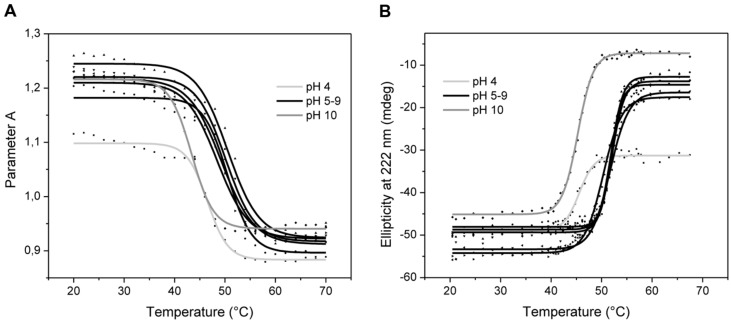
**Variation of ABgp46 tertiary and secondary structure with pH and temperature. (A)** Correlation between fluorescence spectra and conformational changes deduced by plotting Parameter A as a function of the same pH and temperature ranges, for tertiary structure analysis. **(B)** Far-UV Circular dichroism thermal denaturation profiles (from 20 to 70°C, with heating rates of 2°C/min), measured at 222 nm, and at different pH values (pH 4.0–10.0) for secondary structure analysis.

An analysis of the ABgp46 tertiary and secondary structure was performed under different pH values, using CD and protein intrinsic fluorescence, respectively. The fluorescence spectra of ABgp46 obtained at different pH values are depicted in **Figure 2A**. Using the presence of the two tryptophan residues (Trp-95 and Trp-135) in the sequence, an excitation wavelength of 295 nm was used, minimizing the contribution of tyrosine fluorescence. In general, when increasing the temperature from 20 to 70°C, Parameter A showed a similar trend for all pH value and a marked variation was observed at 50°C. Exceptions were pH 4.0 and 10.0 with a Parameter A variation at lower temperatures in the range of 45–46°C. Such spectral variations can be correlated to folding changes of the tertiary structure.

Circular dichroism measurements revealed signal minima around 208 and at 220 nm that prevail in the far-UV CD spectra, which is indicative of α-helices in this protein. The same CD spectra were obtained as a function of pH adjusted from 4.0 to 10.0, indicating an unchanged protein secondary structure (data not shown). To determine the enzyme’s conformational stability in depth, a thermal treatment was employed by increasing temperature of the protein solution at different pH values (**Figure 2B**). Secondary structure changes were analyzed by monitoring CD intensity at 222 nm where α-helix structures show a negative peak. No differences were observed between pH 5.0–9.0, where all T_m_ were recorded in the range of 51–52°C. Extreme pH values of 4.0 and 10.0 were less favorable conditions to maintain the secondary structure integrity, with T_m_ of 44.7 and 44.4°C, respectively. Interestingly, the pH 4.0 was the only condition where the protein did not aggregate after reaching the T_m_, i.e., thermal unfolding. Overall, based on the Parameter A and the ellipticity data, it is likely that tertiary and secondary structure starts to melt at 40°C, resulting in unfolded and denatured protein above 50°C.

### *In vitro* Antibacterial Activity

The *in vitro* antibacterial activity of the recombinant ABgp46 was investigated on a wide set of G^-^ strains (**Table 1**). The activity of Abgp46 alone on tested cells was insignificant as expected, with the exception of *A. baumannii* strains. Interestingly, some *Acinetobacter* strains were mildly sensitive to the endolysin alone, causing between 1 and 2 log reductions of viable cells, including strains resistant to several antibiotics.

**Table 1:** Antibacterial activity of ABgp46 against several Gram-negative bacterial pathogens.

Bacterial species	ABgp46/water	Origin/characteristics
*P. aeruginosa* PAOI	0.03 ± 0.02	Reference strain (ATCC 15692)
*S. typhimurium* LT2	0.14 ± 0.05	Clinical strain
*E. coli* 0157:H7	0.17 ± 0.12	Reference strain (CECT 4782)
*C. sakazakii*	0.23 ± 0.26	Reference strain (CECT 858)
*K. oxytoca*	0.18 ± 0.13	Reference strain (ATCC 13182)
*A. baumannii* #1^∗^	**1.79 ± 0.38**	Clinical strain (TI, AMC, TZP, CE, AZ, ME, AN, GM, NN, CP, PF, TS)
*A. baumannii* #2^∗^	**0.93 ± 0.25**	Clinical strain (TI, AMC, TZP, CE, AZ, ME, AN, GM, NN, CP, PF, TS)
*A. baumannii* #3^∗^	**1.00 ± 0.32**	Clinical strain (TI, AMC, TZP, CE, CEF, AZ, ME, GM, NN, CP, PF, TS)
*A. baumannii* #4^∗^	**0.81 ± 0.21**	Clinical strain (TI, AMC, TZP, CE, CEF, AZ, ME, GM, NN, CP, PF, TS)
*A. baumannii* #5^∗^	0.37 ± 0.19	Clinical strain (TI, AMC, TZP, CE, CEF, AZ, ME, CP, PF)
*A. baumannii* #6^∗^	**1.20 ± 0.46**	Clinical strain (TI, AMC, TZP, CE, CEF, AZ, ME, CP, PF)

*Averages ± standard deviations for all experiments are given for n = 4 repeats. Marked in bold are ≥1 log reduction units observed. ^∗^Known patterns of antibiotic resistance provided by the Hospital of Braga, based on susceptibility tests performed according to the NCCLS for antibiotic resistance; TI, Ticarcillin; AMC, amoxicillin/clavulanic acid; TZP, piperacillin/tazobactam; CE, ceftazidime CEF, Cefepime; AZ, Aztreonam, ME, meropenem; AN, amikacin; GM, gentamicin; NN, tobramycin; CP, ciprofloxacin; PF, Pefloxacin; TS, Trimethoprim/Sulfametoxazol.*

To broaden and increase the antimicrobial effect of the Abgp46 to other G^-^ pathogens, *E. coli* O157:H7 CECT 4782 was chosen as a model strain, to test and optimize an ABgp46/OMP formula. Several organic acids (citric and malic, lactic, benzoic, and acetic acid) were used to sensitize the G^-^ to the endolysin by permeabilizing the OM. Each OMP was used in different concentrations (to achieve three distinct reaction pH values of 7.0, 5.5, and 4.0) and combined with ABgp46 to assess the best antibacterial condition after 2 h incubation (illustrated in **Table 2**). At pH 7.0, only the ABgp46/citric acid was able to slightly reduce *E. coli* cells (0.86 ± 0.28 logs; **Table 2**). At pH 5.5, the effect was broadened to ABgp46/citric acid, ABgp46/malic acid and ABgp46/lactic acid mixtures reducing approximately 1 log of *E. coli* cells (**Table 2**). Consistently, the effect was more pronounced at pH of 4.0. At this pH, the combinatorial effects of ABgp46/citric acid, ABgp46/malic acid, and ABgp46/lactic acid mixtures resulted in 2.78 ± 0.37, 2.08 ± 0.32, and 0.94 ± 0.28 log reductions, respectively (**Table 2**). In this condition, the antibacterial effect was also extended to ABgp46/benzoic acid inactivating approximately 1 log of *E. coli* cells. No effect was observed with ABgp46/acetic acid. Taking into account the most efficient conditions tested, ABgp46/OMPs mixtures (OMPs concentrations used to achieve a pH of 4.0) were tested in the presence of 5 mM MgCl_2_. MgCl_2_ provides an extra source of available divalent cations (Mg^2+^) that can link to the LPS negatively charged phosphate groups, strengthening the LPS monolayer (Vaara, 1999). Therefore, the addition of MgCl_2_ completely abolished the activity of all ABgp46/OMP combinations.

**Table 2:** Combinatorial antibacterial activity of ABgp46 with outer membrane permeabilizers under different concentrations against *Escherichia coli* O157:H7.

pH	OMPs	Molarity (mM)	PBS/OMPs	ABgp46/OMPs	ABgp46/OMPs
					**+ 5 mM MgCI_2_**
7.0 ± 0.1	EDTA	0.50	0.27 ± 0.11	0.32 ± 0.15	
	Citric	0.36	0.21 ± 0.14	**0.86 ± 0.28**	
	Malic	0.60	0.24 ± 0.11	0.53 ± 0.28	
	Lactic	1.20	0.24 ± 0.14	0.55 ± 0.31	
	Benzoic	1.20	0.26 ± 0.15	0.49 ± 0.34	
	Acetic	1.20	0.25 ± 0.14	0.53 ± 0.30	
5.5 ± 0.1	Citric	1.50	0.20 ± 0.13	**1.45 ± 0.25**	
	Malic	3.30	0.17 ± 0.14	**1.09 ± 0.29**	
	Lactic	3.45	0.31 ± 0.07	**0.95 ± 0.14**	-
	Benzoic	3.55	0.27 ± 0.13	0.44 ± 0.33	
	Acetic	4.35	0.26 ± 0.12	0.21 ± 0.22	
4.0 ± 0.1	Citric	3.65	0.28 ± 0.06	**2.78 ± 0.37**	0.15 ± 0.04
	Malic	4.55	0.24 ± 0.18	**2.08 ± 0.32**	0.10 ± 0.03
	Lactic	8.00	0.27 ± 0.18	**0.94 ± 0.28**	0.15 ± 0.04
	Benzoic	10.00	0.57 ± 0.58	**1.11 ± 0.27**	0.02 ± 0.03
	Acetic	20.00	0.22 ± 0.08	0.42 ± 0.29	0.11 ± 0.10

*For each specific pH, different concentrations of EDTA, citric, malic, lactic, benzoic, and acetic acid (given in mM) are used in combination with 2 μM of ABgp46 to reduce E. coli O157:H7 cells in presence or absence of 5 mM of MgCl_2._ Averages ± standard deviations for all experiments are given for n = 4 repeats.Marked in bold are ≥1 log reduction units observed.*

After optimization, citric (at 3.65 mM) and malic (at 4.55 mM) acids were selected as the best OMPs to be combined with ABgp46. Their synergistic effect was further tested on the remaining unsensitized G^-^ strains tested earlier and compared with the gold standard EDTA (at 0.5 mM) OMP (**Table 3**). In the presence of OMPs and ABgp46 no surviving *A. baumannii* cells of strain #2 (phage host strain), could be detected (>5 log reduction; limit of detection, <10 CFU/ml). In addition, *P. aeruginosa* and *S. typhimurium* showed a greater than 4 log reduction when combined with citric or malic acid. Lower, yet still significant reductions, ranging from 1 to 2 logs, were observed against *Cronobacter sakazakii*. In case of *K. oxytoca*, only the ABgp46/citric acid and ABgp46/malic acid combinations showed a moderate effect with log reductions of 0.96 ± 0.35 and 0.81 ± 0.12 viable cells, respectively.

**Table 3:** Combinatorial antibacterial activity of the best ABgp46/outer membrane permeabilizers (EDTA, citric, and malic acid) formula against broad range of planktonic Gram-negative pathogens.

Bacterial species	ABgp46/EDTA	ABgp46/citric	ABgp46/malic
*E coli* O157:H7	0.32 ± 0.15	**2.78 ± 0.37^∗^**	**2.08 ± 0.32^∗^**
*A. baumannii #2*	**>5.00^†^**	**>5.00^†^**	**>5.00^†^**
*P. aeruginosa* PAOI	**4.35 ± 0.20**	**4.25 ± 0.31**	**4.51 ± 0.22**
*S. typhimurium* LT2	0.57 ± 0.20	**4.24 ± 0.39^∗^**	**4.07 ± 0.37^∗^**
*C. sakazakii CECT 858*	**1.21 ± 0.38**	**2.05 ± 0.27**	**0.94 ± 0.23**
*K. oxytoca A1CC* 13182	0.26 ± 0.08	**0.96 ± 0.35^∗^**	0.81 ± 0.12^∗^

*Averages ± standard deviations for all experiments are given for n = 4 repeats. Marked in bold are ≥1 log reduction units observed. Statistically significantly (Student’s t-test and P < 0.05) conditions between ABgp46/EDTA and ABgp46/organic acids are indicated with asteristic. ^†^The detection level of 10 CFU/mL has been reached.*

Overall, an antibacterial effect was observed when ABgp46 was combined with different OMPs, and its effect was found to be more pronounced in the presence of citric or malic acid than when the chelating agent EDTA was used.

## Discussion

G^-^ bacterial infections have always been a threat to human health. In particular, infections caused by antibiotic resistant bacteria are problematic and their incidence is constantly reported worldwide (Chopra et al., 2008; Scallan et al., 2011). Phage-encoded endolysins represent one promising avenue of investigation to fight these pathogens.

Biochemical characterization assays were carried out with ABgp46 to expand the knowledge of the G^-^-like endolysins activity and their conformational stability. LC-MS experiments demonstrated that ABgp46 is an *N*-acetylmuramidase, cleaving the PG between MurNAc and GlcNAc glycan strands. Compared to previously reported G^-^-like globular endolysins (Supplementary Table S1), ABgp46 has a similar muralytic activity, and is more active at alkaline conditions (pH 8.0–9.0; Lai et al., 2011; Walmagh et al., 2013). Like others (Supplementary Table S1), ABgp46 also retains its activity once refrigerated for at least 1 month, and only few endolysins are regarded as heat-resistant proteins (Walmagh et al., 2012, 2013). The biochemical characterisation of the endolysin was also complemented by analyzing the stability of its structure. In this study, we employed fluorescence and CD studies that indicated that ABgp46 has a melting temperature of 52°C, at the optimal pH value. The good correlation between muralytic activity and conformational changes as a function of temperature strongly suggests that the decrease of activity observed for ABgp46 above 50°C is mainly due to structural protein modifications. Similar studies have only been conducted with a *Salmonella* phage endolysin Lys68, with an observed melting temperature of 44°C (Oliveira et al., 2014).

To evaluate the potential use of ABgp46 as an antimicrobial compound, the enzyme was first tested without the addition of a permeabilizer. Interestingly, despite the present of the OM protecting the PG layer, externally added ABgp46 was active against several multi-resistant *Acinetobacter* strains (14 out of 17 antibiotics). This is a rare event reported for G^-^-like endolysins. The T4 lysozyme (from the T4 phage), was the first endolysin described to naturally kill G^-^ cells. It was shown that its C-terminal positively charged amphipathic α-helix (named α-4) is responsible to cause OM disruption, having a stronger bactericidal effect than the enzymatic PG hydrolysis (During et al., 1999). Later, four other endolysins (OBPgp279, Lys1521, PlyF307, and LysAB2) were also reported to spontaneously inactivate G^-^ cells (Morita et al., 2001; Lai et al., 2011; Walmagh et al., 2012; Lood et al., 2015). However, opposite to the bactericidal effect of synthetic α-4 of T4 lysozyme, it is assumed that the C-terminal positively charged region of these endolysins does not have an antibacterial effect *per se*, but rather mediate the N-terminal enzymatic domain to enter the cells, allowing them to digest the PG and cause bacteriolysis. In general, these endolysins capable of spontaneously killing G^-^ bacteria show a very specific antibacterial activity. This intrinsic antibacterial activity that was also observed for ABgp46 can be explained by the same C-terminal amphipathic region (amino acid sequence between 112 and 145) identified in LysAB2.

To improve the antibacterial activity, ABgp46 was combined with concentrations of OMP agents (citric, malic, lactic, benzoic, and acetic acid), reaching maximum activity at pH of 4.0. It is known that some weak organic acids (e.g., citric, malic, benzoic, and lactic acid) have, to a lesser extent, chelating properties, but the additional acidity can also contribute to OM disruption (Alakomi et al., 2000; Theron and Lues, 2011). LPS disintegration is accomplished by the ability of undissociated acid groups to interact/pass through the negatively charged LPS (whereas negatively charged acid forms are repulsed) and migrate inside the cells to cause sub-lethal injuries. Intracellularly, the organic acid meets a higher internal pH (pH 7.2) and dissociates to produce protons (that can exit through specific proton channels) and anions (Breidt et al., 2004). Because the following bactericidal decreasing effect was observed: citric acid (pKa 3.13) > malic acid (pKa 3.4) > lactic acid (pKa 3.86) ≈ benzoic acid (pKa 4.19) > acetic acid (pKa 4.76) when combined with ABgp46, we speculate that this it is a reflection of acid dissociation constant (pKa) increase. This is somehow unexpected, since increasing the pKa favors undissociated groups that could penetrate into the cytoplasmic membrane potentiating cell damage. In contrast, acids with low pKa values would produce more hydrogen ions (that are not able to internalize) when exposed to an aqueous environment. Nevertheless, a similar trend was observed when organic acids (with lower pKa values) were incubated with medium-chain fatty acids (Kim and Rhee, 2013). These authors hypothesized that the medium-chain fatty acid action interact with the bacterial cell membranes, causing sub-lethal injuries. This would lead hydrogen ions to pass into the cell and result in a marked bactericidal effect. Perhaps a similar mechanism explains why ABgp46/citric acid and ABgp46/malic acid are more powerful combinations compared to other OMPs. The fact that no bacterial effect is observed in presence of 5 mM of MgCl_2,_ also suggests that free magnesium ions strengthen the electrostatic interactions between neighboring LPS components, avoiding acid entry and sequential sublethal damage (Vaara, 1999).

From preliminary testing, citric acid and malic acid were chosen for further testing against a larger group of G^-^ cells and compared with EDTA. ABgp46/EDTA revealed to be the only combination efficient against *Pseudomonas* and *Acinetobacter* cells, the same pattern was previously observed with Lys68 (Oliveira et al., 2014). This can be explained by the high phosphate content and consequently a higher concentration of stabilizing divalent cations present in the OM of these bacteria, compared with *Enterobacteriaceae* (such as *E. coli* and *S. typhimurium*; Nikaido, 2003), that are therefore, more prone to a chelation effect. The antibacterial effect of ABgp46 is more broaden in the presence of organic acids that are predicted to destabilize the bacterial OM by an acidic effect, probably by lowering the bacterial internal pH and the accumulation of toxic substances (sub-lethal injury; Alakomi et al., 2000; Theron and Lues, 2011).

In summary, the structural and enzymatic characterization of ABgp46 provides novel insights into G^-^-like endolysins, and indicated that ABgp46 is an effective endolysin against several multidrug resistant pathogens. This technology can be appealing as a therapeutic/disinfectant agent for a range of applications. For instance, the use of topical solutions (e.g., cream or lotions) containing the endolysin/OMP formula could be useful for treating skin and soft tissue infections, associated with acne or chronic wounds.

## Author Contributions

LK, FS, and JA conceived the study. LK, RL, SS, and JA analyzed data. HO, DV, and SM performed experiments. HO and SM wrote the paper.

## Conflict of Interest Statement

The authors declare that the research was conducted in the absence of any commercial or financial relationships that could be construed as a potential conflict of interest.
