# Supplementary material for: Structural and Enzymatic Characterization of ABgp46, a Novel Phage Endolysin with Broad Anti-Gram-Negative Bacterial Activity
Source: Front Microbiol. 2016 Feb 26;7:208. doi: 10.3389/fmicb.2016.00208 (PMC4768612; doi:10.3389/fmicb.2016.00208)
Supplement: Supplementary file 1 [file Data_Sheet_1.DOCX]

**Table S1.** **Comparison of the biochemical characteristics of endolysins from phages infecting Gram-negative hosts.** For each endolysin ABgp46 (KP998152), OBPgp279 (YP_004958186), PVPSE1gp146 (YP_004893953), P2gp09 (NP_046765), BcepC6Bgp22 (YP_024942.1), Lys68 (KJ475444), K11gp3.5 (YP_002003804)) and KP32gp15 (YP_003347533.1), the respective accession number, phage host, predicted structure (globular/modular and respective domains), muralytic activity (expressed in units/μM), pH optimum and stability data is given. All endolysins were characterized using equal *Pseudomonas* PG substrates and biochemical methodologies.

Catalytic domain acronyms: GH19 (PF00182) and GH24 (PF00959) are glycoside hydrolases of the family 19 and 24, respectively. Ami-2 (PF01510) is an amidase that cleaves the PG bond between the N-acetylmuramoyl residues and L-amino acid residues. Binding domain acronym: PG-1 (PF01471) is a peptidoglycan binding domain rarely observed in G^-^-acting phage endolysins.

**Figure S1.** **Analysis of ABgp46 enzymatic activity.** RP-HPLC analysis of *E. coli* BW25113 Δ*lpp* peptidoglycan fragments solubilized by *S. globisporus* mutanolysin (muramidase), *E. faecalis* AtlA (*N*-acetylglucosaminidase) and recombinant ABgp46..

**
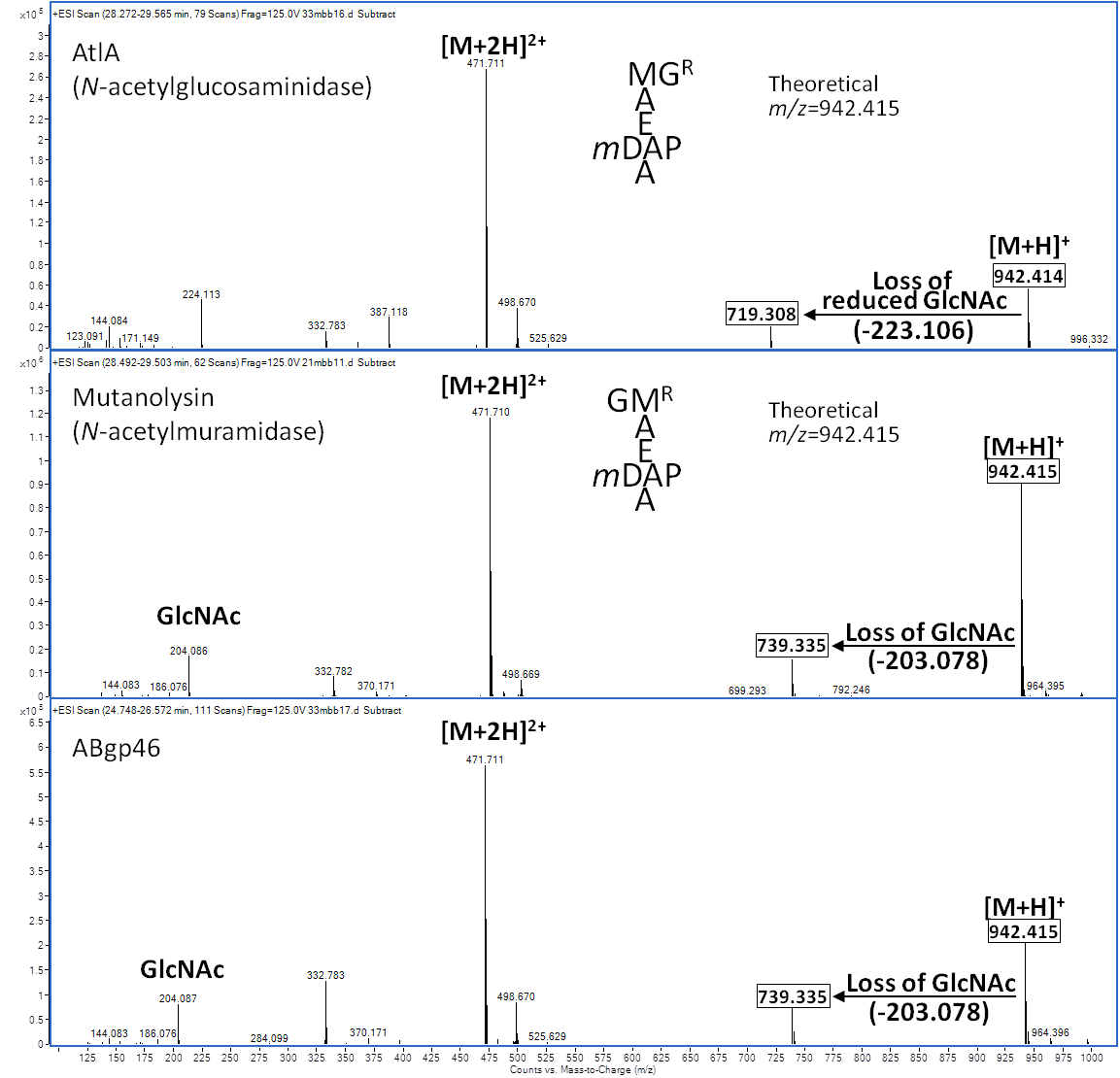
**

**Figure S2. Determination of ABgp46 cleavage specificity.** LC-MS analysis of the major disaccharide-peptides in peaks 1, 2 and 3. Major ions in peaks 1 (mutanolysin digestion) and 3 (ABgp46 digestion), show a fragmentation event leading to the loss of a non-reduced GlcNAc residue (loss of 203.078 atomic mass units, seen as a [M+H]^+^ adduct with an *m/z* at 204.086 and 204.087) indicating that ABgp46 displays *N*-acetylmuramidase (lysozyme) activity. By contrast, the major ion in peak 2 (AtlA digestion) shows a fragmentation event leading to the loss a reduced GlcNAc residue (loss of 223.106 atomic mass units, seen as a [M+H]^+^ adduct with an *m/z* at 224.113) expected for an *N*-acetylglucosaminidase activity. The sequence of muropeptides is indicated together with their respective *m/z* values (*boxed*). *A*, L-Ala or D-Ala; *a*, C-terminal D-Ala; *m-DAP*, *meso*-diaminopimelic acid; *E*, g-D-Glu; *M^R^*, reduced *N*-acetylmuramic acid; *G*, *N*-acetylglucosamine; *G^R^*, reduced *N*-acetylglucosamine.

**REFERENCES**

Oliveira, H., Thiagarajan, V., Walmagh, M., Sillankorva, S., Lavigne, R., Neves-Petersen, M.T., et al., (2014). A thermostable *Salmonella* phage endolysin, Lys68, with broad bactericidal properties against gram-negative pathogens in presence of weak acids. *PLoS One*. 9, e108376. doi: 10.1371/journal.pone.0108376.

Walmagh, M., Boczkowska, B., Grymonprez, B., Briers, Y., Drulis-Kawa, Z., Lavigne, R., (2013). Characterization of five novel endolysins from Gram-negative infecting bacteriophages. *Appl Microbiol Biotechnol*. 97, 4369-4375. doi: 10.1007/s00253-012-4294-7.

Walmagh, M., Briers, Y., dos Santos, S.B., Azeredo, J., Lavigne, R., (2012). Characterization of modular bacteriophage endolysins from *Myoviridae* phages OBP, 201phi2-1 and PVP-SE1. *PLoS One*. 7, e36991. doi: 10.1371/journal.pone.0036991.
